# Recent advances in our understanding of the allograft response

**DOI:** 10.12703/r/10-21

**Published:** 2021-02-25

**Authors:** Conor Hennessy, Guido Lewik, Amy Cross, Joanna Hester, Fadi Issa

**Affiliations:** 1 Transplantation Research Immunology Group, Nuffield Department of Surgical Sciences, University of Oxford, John Radcliffe Hospital, Oxford, OX3 9DU, UK

**Keywords:** transplantation, rejection, allorecognition, advances

## Abstract

Organ transplantation is a life-saving treatment for end-stage organ failure. However, despite advances in immunosuppression, donor matching, tissue typing, and organ preservation, many organs are still lost each year to rejection. Ultimately, tolerance in the absence of immunosuppression is the goal, and although this seldom occurs spontaneously, a deeper understanding of alloimmunity may provide avenues for future therapies which aid in its establishment. Here, we highlight the recent key advances in our understanding of the allograft response. On the innate side, recent work has highlighted the previously unrecognised role of innate lymphoid cells as well as natural killer cells in promoting the alloresponse. The two major routes of allorecognition have recently been joined by a third newly identified pathway, semi-direct allorecognition, which is proving to be a key active pathway in transplantation. Through this review, we detail these newly defined areas in the allograft response and highlight areas for potential future therapeutic intervention.

## Introduction

Allotransplantation saves and improves many thousands of lives every year and is the most common treatment for end-stage lung, heart, liver and kidney disease. The global demand continues to rise, yet there are unresolved challenges to this therapy. Tissue typing, organ preservation and immunosuppressive drugs have facilitated the survival of more than 80% of solid organ transplants at two years post-transplantation. However, lifelong immunosuppression has its drawbacks, and patients are susceptible to a host of issues, including infections^1^, nephrotoxicity^2^ and cancer^3^. Ultimately, tolerance in the absence of immunosuppression is the goal, and the ideal scenario results in weaning the patient gradually from immunosuppression with no adverse effects. Tolerance seldom occurs, however, with removal of immunosuppression invariably resulting in graft destruction by the host immune system. There is therefore a need for novel methods of immunotherapy to circumvent the challenges with current immunosuppression. The development of these novel therapeutics requires an in-depth understanding of the alloresponse.

The alloresponse is the term used to describe the response of the immune system to cells of non-self origin. It is divided into two components: the first is allorecognition, concerning the recognition of non-self antigens by cells of the host’s immune system; the second is the ensuing destructive effector response^4^. The traditional T- and B-cell responses to the allograft have been well reviewed (for example, 5). This review therefore will explore the more recent advances in our understanding of the alloresponse and focus on innate lymphoid cells (ILCs), natural killer (NK) cells, and novel pathways of allorecognition.

## Innate lymphoid cells

**Figure 1.**
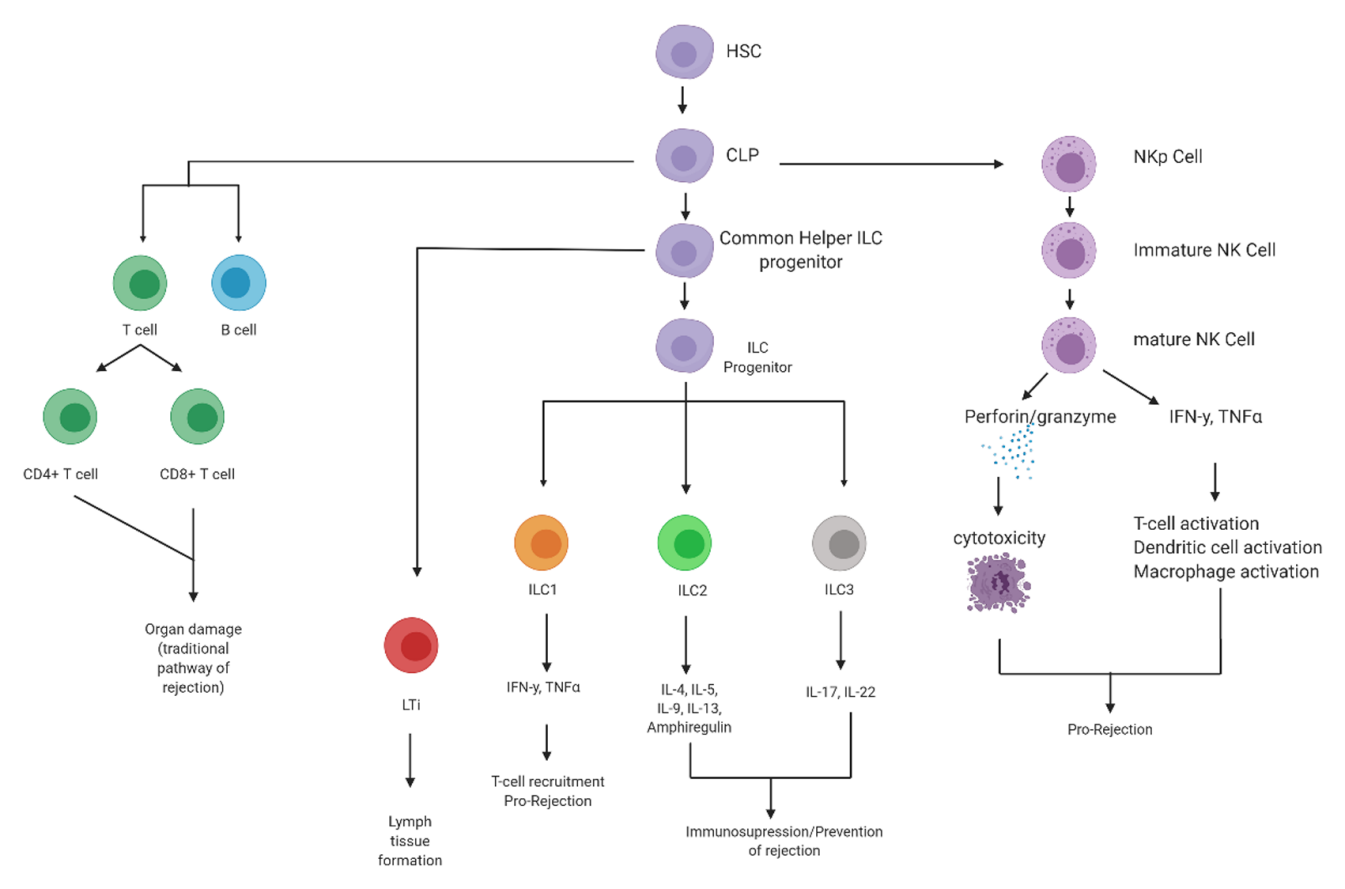
Lineage of innate lymphocytes and their proposed roles in alloresponses. Until recently, it was believed that the common lymphoid progenitor (CLP) was the common ancestor for only the innate natural killer (NK) cells and lymphoid tissue-induced cells as well as the T and B cells of the adaptive immune system. However, in the last decade, a further subgroup of innate lymphocytes, termed innate lymphoid cells 1–3 (ILC1–3), was discovered. Emerging research suggests that NK cells may have a significant role in the alloresponse and allograft rejection through direct cytotoxic actions and activation of T cells, dendritic cells and macrophages. The newly classified ILCs are believed to have mixed roles in the alloresponse. ILC1 cells are thought to be pro-inflammatory, acting via interferon gamma (IFNγ) and tumour necrosis factor alpha (TNFα) to recruit T cells and increase inflammation propagating organ rejection. ILC2 and ILC3 cells are believed to act in an immune-regulatory manner, acting via cytokines and secreted factors to prevent allograft rejection. Figure created using BioRender. HSC, haematopoietic stem cell; IL, interleukin; LTi, lymphoid tissue inducer cells; NKp, natural killer precursor.

The common lymphoid progenitor (CLP) is the developmental precursor for the effector cells of the adaptive immune response: T cells, B cells, and the ILCs which are devoid of any genetically rearranged antigen specificity (Figure 1). NK cells were the first of these ILCs to be defined and are commonly viewed as the non-specific counterpart to CD8^+^ T cells. These cells are instrumental to the destruction of transformed, infected or stressed cells^6^. The second group to be described consisted of lymphoid tissue inducer cells, whose function was determined to induce the development of lymph nodes and Peyer’s patches^7,8^. In the past decade, however, a new subset of these ILCs that developmentally and phenotypically resemble CD4^+ ^T helper (Th) cells has been classified. They share a common lineage with T cells, B cells and the other innate lymphocytes, all of which are the progeny of the CLP (Figure 1). It is believed that, following the divergence with the T-cell and B-cell lineages, the CLP gives rise to a specific ILC progenitor termed the common innate lymphoid progenitor^9^. ILCs, rather than responding to specific antigens, have key roles in defence against infection and wound healing and are activated by microbial compounds, stress responses and the inflammatory environment of surrounding tissues^10–12^. Unlike their adaptive counterparts, ILCs do not express specific antigen receptors, and as such their immune action is largely non-specific. However, similarities between ILCs and T cells in terms of cytokine production have led to ILCs being classed as the innate counterparts of T cells. These ‘novel’ ILCs therefore have been subclassified on the basis of their ability to produce Th2-associated cytokines (interleukin 5 [IL-5] and IL-13), Th17-associated cytokines (IL-17 and IL-22) or interferon gamma (IFNγ)^12^. Group 1 ILCs (ILC1s) are defined by the production of IFNγ and the inability to produce Th17- and Th2-associated cytokines. Group 2 ILCs (ILC2s) are defined by their production of Th2-associated cytokines, and group 3 ILCs (ILC3s) are defined by their ability to produce Th17-associated cytokines. Studies have demonstrated that developing ILCs are generally bone marrow-resident in the foetus and liver-resident in adults but that mature ILCs are normally tissue-resident, mainly in the liver, lungs, gastrointestinal tract and skin^13–15^. Until recently, very few studies have examined the role of ILCs in graft tolerance and rejection, but studies published in recent years have started to highlight the potential importance of these cell types in the allograft response.

### Innate lymphoid cells in lung transplantation

Recent studies have suggested a key role for ILCs in the alloresponse following solid organ transplantation. Monticelli *et al*. determined that changes in the levels of certain ILCs were associated with the development of allograft dysfunction following lung transplant^16^. The single-centre cohort study determined that the development of primary graft dysfunction (PGD), a form of acute lung injury that occurs after lung transplantation, was associated with decreased populations of ILC2 cells within the donor lung after reperfusion. This decrease was not observed in the non-PGD cohort. The study also showed that in participants who did not develop PGD, there was an increase in NCR^−^ ILC1 cells (but no change in NCR^+^ ILC1 cells) in pre-perfusion samples, and no such change was observed in the PGD cohort. This suggests that the composition of ILCs pre- and post-perfusion may influence the development of PGD. Previous mouse models had showcased a pro-inflammatory role for ILC2 cells in lung disease, acting mainly via IL-13 and IL-5 to propagate airway hyper-reactivity^17–21^, but an earlier article by Monticelli *et al*. demonstrated that in mouse models of severe lung injury, ILC2 cells were instrumental in promoting airway repair and remodelling through the growth factor amphiregulin, a key mediator of tissue repair and remodelling which promotes epithelial cell and fibroblast proliferation^22^. This role for amphiregulin in airway remodelling post-transplantation was further demonstrated in a recent study by Todd *et al*., where amphiregulin was shown to be associated with fibrosis and lung repair in chronic lung allograft dysfunction^23^. The 2020 study by Monticelli *et al*. also determined that changes in the subset composition of ILC1 cells may be implicated in the rate of PGD, showing that there was an increased proportion of NKp44-negative ILC1s (NCR^−^) in the biopsies of patients without PGD when compared with those who developed PGD^16^. The authors suggest that this ‘less active’ subset of ILC1 cells may be associated with protection from reperfusion-associated graft injury, in contrast to Nkp44-positive ILC1 cells, which have been associated with graft damage and organ rejection.

Recent work by Tanaka *et al*. identified a role for ILC3 cells in lung allograft survival, with ILC3-derived IL-22 thought to facilitate tolerance^24^. This study demonstrated that ILC3 cells, along with γδ T cells, produce IL-22, an essential inducer of bronchus-associated lymphoid tissue (BALT) formation in allografts. Previous studies have suggested that the formation of BALT following lung transplantation is associated with chronic rejection^25^. However, more recent work has suggested that the formation of BALT plays a key role in the maintenance of tolerance^26,27^.

### Innate lymphoid cells in intestinal transplantation

Type 3 ILCs (ILC3s) have been associated with better outcomes after intestinal transplantation. A 2020 study identified the balance of ILC1/ILC3 cells within the grafts after transplantation as correlating with graft function and survival. When unsuccessful transplants were compared with healthy grafts at 6 months, it was found that there was a decrease in ILC3 numbers in the failed transplants^28^. The authors determined that immediately following transplantation, the population of ILC3 cells was decreased in all grafts but that the population recovered within 2 to 4 weeks in healthy grafts and failed to recover in unsuccessful allografts. This suggests a key role for ILC3s in the maintenance of tissue homeostasis following intestinal transplant. The authors posit that the production of IL-22 by ILC3s is the main mechanism by which they support graft survival. Earlier studies on the role of IL-22 in intestinal transplant survival suggested that IL-22 increases proliferation and expansion of intestinal stem cells post-transplantation, which results in improved mucosal regeneration and epithelial repair^29^. However, the relationship between ILCs and graft survival is not as clear-cut, as in the case of the study by Kang *et al*., there was an increase in numbers of ILC1 cells in unsuccessful grafts^28^. These ILC1s act to produce pro-inflammatory IFNγ and tumour necrosis factor alpha (TNFα); the implication is that in the absence of the protective effects of the IL-22-producing ILC3 cells, the increased ILC1s promote graft dysfunction and rejection.

### Innate lymphoid cells in graft-versus-host disease

Allogenic bone marrow transplantation is a mainstay of management in many haematological malignancies and immunological pathologies, including lymphoma, myelodysplasia, myeloma and leukaemia^30^. The curative potential of haematopoietic stem cell transplantation (HSCT) is often complicated by the risk of graft-versus-host disease (GvHD), in which donor T cells target host alloantigens, resulting in the production of a number of inflammatory cytokines^31^. CD4^+^ T cells produce Th1 cytokines (IFNγ, TNFα and IL-1) following activation by antigen-presenting cells (APCs), which act locally and systemically to potentiate the effects of alloreactive T cells. CD4^+^ and CD8^+^ donor T cells also exhibit antigen-specific cytotoxicity in GvHD, and the secretion of perforin and granzyme causes cell lysis and apoptosis. Studies estimate that between 35% and 50% of HSCT recipients will experience acute GvHD and that 50% of these will progress to chronic GvHD^32^. The prognosis can be quite poor; 30% long-term survival is seen in grade 3 GvHD and only 5% survival in grade 4 GvHD.

Emerging evidence suggests that ILCs have a key role in regulating the CD4^+^ and CD8^+^ T-cell responses that govern the graft-versus-host response in HSCT. Studies have suggested that ILC3s exert a protective function via IL-22 secretion^33^, similar to previously discussed intestinal transplant studies. This protective role for IL-22 has been observed in mouse models, and the absence of IL-22 in models of GvHD is associated with much more severe disease^34^. The use of recombinant IL-22 in humans who are experiencing acute GvHD is being evaluated in clinical trials (ClinicalTrials.gov Identifier: NCT02406651), and results are expected soon. ILC2 cells are also believed to have a protective role in GvHD. It is thought that ILC2s exert their effects not via a cytokine-secreting mechanism such as that seen with ILC3s, but through the recruitment of myeloid-derived suppressor cells and also via suppression of Th1 and Th17 cells^35^. Th1 and Th17 cells have been established as key effectors of the alloresponse that precipitate GvHD^36,37^, and ILC2-derived IL-13 and amphiregulin are capable of inhibiting their recruitment^35^.

### The future of innate lymphoid cells and the alloresponse

Although there is a paucity of data regarding the role of ILCs in alloimmunity and the alloresponse, the studies that have been reported suggest a key role for this recently classified group of cells in regulating alloresponses in transplantation. Further understanding of the exact mechanisms by which these cells mediate the alloresponse is needed. The clinical trial examining the role of IL-22 in treating acute GvHD is an indicator of the potential for ILC-related therapies, yet there are many challenges to overcome, not least the apparent plasticity of ILCs. Studies have suggested that in the presence or absence of certain cytokine signals, ILCs can undergo type switching, in which IL-13- and IL-22-producing ILC2s and ILC3s convert to pro-inflammatory IFNγ-producing ILC1s^38–41^. Therapies that seek to expand the population of regulatory ILCs and prevent them from transdifferentiation could provide potential for future therapies. The administration of recombinant factors produced by the ILCs could also provide therapeutic benefit. Administration of IL-22 is already under investigation, and amphiregulin and IL-13 are also of interest.

## Natural killer cells

NK cells were the earliest discovered members of the now termed ILC population and are so called for their innate or ‘natural’ ability to kill cells without the need for prior exposure^42^. Self-recognition of class I major histocompatibility (MHC) via inhibitory killer cell immunoglobulin-like receptors (KIRs) prevents autologous cell destruction, although NK cells have a low threshold for detection of deficient or altered MHC I expression such that may be seen in certain tumours, allowing the detection of mutated cells where necessary^43^. Based on the missing-self hypothesis, the assumption is that NK cells would target transplanted organs and therefore be key players in the alloresponse. Although there was evidence that NK cells played a role in HSCT rejection^44^, there was little evidence that NK cells had any role in solid organ rejection. This theory was reinforced by studies which examined the potential role of NK cells in graft rejection, and depletion of NK cells in models of skin graft rejection and cardiac transplant rejection failed to prevent or delay graft rejection^45–47^. Thus, despite the intrinsic ability of NK cells to recognise the ‘non-self’, their contribution to allograft rejection was considered negligible.

The last decade, however, has provided new insights into the role of NK cells in solid organ rejection. A 2017 study by Kawakami *et al*. suggested a key role for NK cells in bronchiolitis obliterans (BO), a form of chronic rejection seen after lung transplantation^48^. In a mouse model of BO, a much higher percentage of NK cells was identified in allografts when compared with isografts, and anti-NK1.1 depletion of NK cells attenuated rejection. Furthermore, inhibition of NKG2D, an NK cell activating receptor, was found to inhibit the development of BO. However, it is worth considering that the NKG2D receptor is also constitutively expressed on CD8^+^ T cells, and studies suggest it is a co-stimulatory receptor that activates T cells alongside the T-cell receptor^49^. Thus, it is possible that the effect seen with NKG2D blockade is at least partially related to T-cell inhibition. Furthermore, the study by Kawakami *et al*.^48^ used a heterotopic tracheal transplant model of BO, which has limitations in modelling immune events after transplantation^50^.

A 2019 study by Koenig *et al*. challenged the established paradigm of antibody-mediated microvascular inflammation (MVI) as the main cause of graft failure, suggesting that NK cells may instead trigger MVI in an antibody-independent manner^51^. The authors demonstrated that graft endothelial cells (ECs) were capable of activating recipient NK cells because of the mismatched HLA class I molecules^51^. The study showed that the graft ECs triggered the ‘missing self’ based activation of NK cells which resulted in MVI. NK-associated MVI was shown to act via the mTORC1 pathway in NK cells, and rapamycin, an mTORC1 inhibitor, could inhibit the development of this NK cell-mediated MVI. Importantly, this study demonstrates a non-antibody-mediated mechanism for MVI in graft injury, which was previously believed to be solely due to antibody-mediated rejection and which potentially is controlled with the use of mTORC1 inhibitors.

In another landmark article examining the role of NK cells in organ rejection, Niehrs *et al*. showed that NK cells are capable of detecting HLA class II molecules, namely HLA-DP subsets via the NK cell receptor NKp44^52^. The ability of NK cells to detect HLA class II molecules gives insight into recent research that demonstrated an increased risk of GvHD in individuals who possess a high-expression allele of HLA-DP^53^. Furthermore, the study by Niehrs *et al*.^52^ demonstrated that the NK cells interacted with the HLA-DP only via NKp44, an NK activating receptor which is expressed only by activated NK cells, such as in infection, graft inflammation and rejection^54^. Interestingly, this study also showed that the use of anti-NKp44 antibodies could prevent NK-APC association^52^, suggesting that direct NK inhibition may prevent the Nkp44-mediated alloresponse. Wu and Li remarked that since MHC II is usually expressed by professional APCs, NK cells may be interacting with APCs, both host- and donor-derived, in the transplant setting^54^. Thus, NK cells could be acting to increase T-cell priming and activation, causing graft injury through direct cytotoxic mechanisms or, as one study has suggested, causing a switch in the method of donor antigen presentation from the direct to indirect allorecognition^55^. Applying this evidence of NK activity in transplantation and working towards a therapeutic angle, Ashraf *et al*. showed that depletion of NK cells in combination with cyclosporin treatment (in a renal transplant model in mice) results in enhanced transplant survival together with the development of a Foxp3^+^ regulatory T (Treg) population^56^.

KIRs are cell surface receptors present on NK cells and are classified as activating or inactivating KIRs depending on whether they express an activating or inhibiting tyrosine kinase^57^. Although KIRs are known to play a role in many inflammatory conditions, until recently there was conflicting evidence regarding their role in transplant rejection^58–62^. A 2017 study by Littera *et al*. demonstrated a key role for KIRs in chronic rejection following kidney transplantation^63^. The study identified that when recipient-donor pairs lacked two specific functional KIR-HLA ligand combinations (rKIR2DL1/dHLA-C2 and rKIR3DL1/dHLA-Bw4), they were at significantly higher risk of organ rejection^63^. This immunogenetic profile resulted in significantly lower levels of NK inhibition and suggested that KIR genotyping prior to transplantation may be a useful measure in the future and that there is a potential role for direct anti-NK immunotherapy in this setting.

Studies are also beginning to demonstrate a role for NK cells in tolerance. Perhaps one of the earliest studies to identify a role for NK cells in tolerance was in 2005, when NK cells were shown to promote tolerance in islet allografts by interacting with recipient immune cells^64^. This study suggested that NK cells promoted tolerance by inhibiting CD4 and CD8 T cells, as well as DCs, in a perforin-dependent manner. A subsequent study highlighted a pro-tolerogenic role for NK cells in skin allograft tolerance, showing that NK cells impede the actions of donor-derived APCs, resulting in decreased alloreactive T-cell activation^65^. A 2013 study echoed these findings, demonstrating a pro-tolerogenic role for NK cells in a mouse model of allogenic lung transplantation^66^. Here, NK cells infiltrated lung allografts and inhibited T-cell infiltration, resulting in decreased alloreactive T-cell activation and increased graft survival. Host NK cells inhibited T-cell activation through the destruction of donor-derived DCs in a manner similar to that outlined by Yu *et al*.^65^. An intriguing role for host NK cells has also been identified in alloantibody responses, whereby host NK allorecognition and killing of donor CD4 T cells prevent the latter’s ability to provide help to host B cells^67^. The role of NK cells in transplantation is therefore not straightforward, and there is evidence that these cells may act in both directions to promote or prevent transplant rejection.

## The adaptive alloresponse

**Figure 2.**
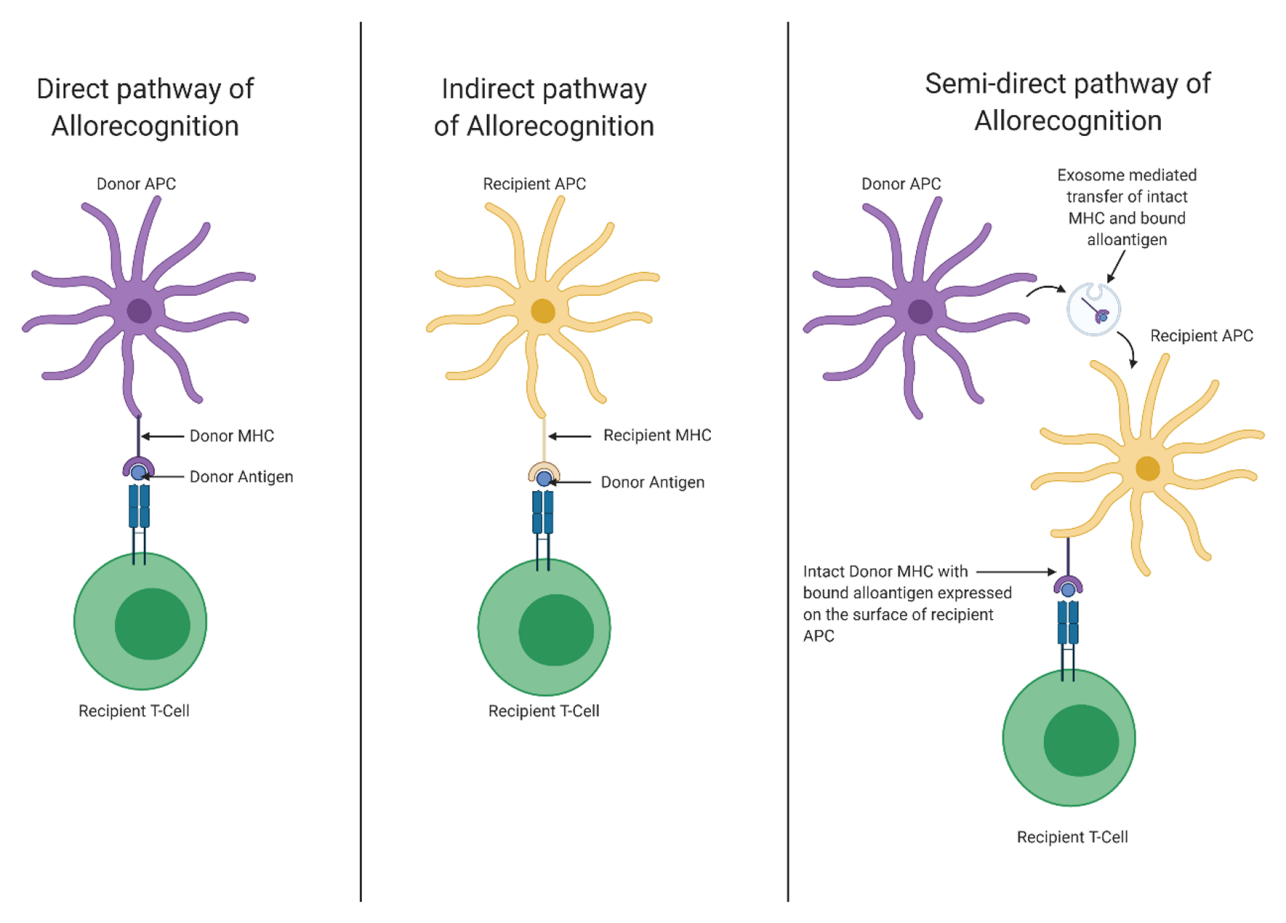
The pathways of allorecognition. The direct pathway of allorecognition involves the recognition of donor antigens by recipient T cells, which bind to donor major histocompatibility molecules (MHCs) on the surface of donor antigen-presenting cells (APCs). The indirect pathway involves the presentation of processed donor antigens by MHC molecules on recipient APCs to recipient T cells. Until recently, these were believed to be the only two pathways involved in allorecognition. However, evidence has emerged for a third pathway, termed semi-direct allorecognition, in which intact donor MHC-antigen complexes are presented by recipient APCs to recipient T cells. Figure created using BioRender.

The central hypothesis in allograft rejection is based on T-cell recognition of donor MHC proteins. Traditionally this allorecognition was believed to occur via two mechanisms: the direct pathway and the indirect pathway^68^. When acting via the direct pathway, recipient T cells recognise donor (graft) MHC molecules on the surface of donor APCs to trigger the alloresponse, which is thought to principally contribute to acute organ rejection. The indirect pathway is mediated by host APCs uptaking and processing donor MHCs with subsequent presentation to alloreactive T cells. However, recent evidence suggests that a third pathway, semi-direct allorecognition, is highly relevant in transplant rejection (Figure 2).

Semi-direct allorecognition refers to the pathway of T-cell allorecognition of host APCs which have acquired intact donor antigen (usually MHC molecules). Classically, it was thought that following organ transplantation donor dendritic cells (DCs) migrated as ‘passenger leukocytes’ from the graft to host lymph tissues, wherein they presented donor MHC molecules to alloreactive T cells^69,70^. This theory has little evidence to support it and donor DCs are absent or found in only very low numbers in allografts a week after transplantation^71,72^. This lends little credence to the theory that donor-derived DCs are the driving force of alloreactive T-cell activation in the early stages of rejection. In fact, recent mouse studies have demonstrated that intact donor MHCs can exist on the surface of host APCs, and these so-called ‘cross-dressed’ APCs can present the donor MHC molecules directly to host T cells^70,73^.

The transfer of antigen between different cell types is not novel; studies show that the leukocytes can transfer surface antigens, including MHC I and II, through a number of mechanisms termed cross-dressing, cell-nibbling or trogocytosis^74–76^. This concept of cross-dressing was originally proposed by Lechler *et al*. in 2004^77^; following this seminal article, studies have identified recipient APCs which expressed intact donor MHCs in graft-draining lymph tissues following mouse kidney and heart transplantation^78,79^. Although there has been evidence for this semi-direct pathway for just over a decade, the mechanism by which APCs acquire donor MHC molecules has only recently begun to be elucidated.

Cell-to-cell contact via receptor-ligand interactions and the release of soluble mediators such as cytokines and extracellular vesicles (EVs) are key mediators of inter-cellular communication^80^ alongside established cell-cell communication mechanisms. The term EV encompasses microvesicles, exosomes (nanovesicles), apoptotic blebs and other yet-to-be-classified EVs^81,82^. Exosomes function as carriers for nucleic acids, proteins and possibly lipids and carbohydrates between cells, and the composition of the exosome is dependent on the lineage and function of the cell it is derived from^73^. This carrier function of exosomes would suggest that they may have a role in antigen trafficking between APCs; indeed, emerging research suggests that the cross-dressing of recipient APCs occurs via an exosome-mediated transfer of intact donor MHC molecules from donor APCs, which may act as the main driver of acute T-cell activation following allotransplantation^73^.

A 2020 study by Hughes *et al.* underscores the importance of cross-dressed host DCs in mouse islet and kidney transplant models^83^. Semi-direct allorecognition was found to drive the early allograft response in secondary lymphoid organs as well as the graft itself, proving sufficient to mediate acute rejection within 26 days^83^. Conversely, a loss of recipient DCs resulted in prolonged graft survival. This mirrors previous data which showed replacement of passenger leukocytes by recipient DCs soon after transplantation and a drastic decrease in overall donor MHC presentation upon selective recipient DC depletion^71,84^. Yet, as early as 2 months after engraftment, cross-dressed recipient DCs appeared to vanish from the allograft^83^. While this could indicate the transience of this response pathway, Smyth *et al.* previously showed that after donor leukocyte removal, acquired allo-MHC I-peptide complexes on recipient DCs can drive allograft rejection throughout the lifespan of the transplant^85^. Further evidence comes from studies using Batf3^−/− ^mice (which lack CD8α^+^ conventional DCs), where induction of the alloresponse following skin transplantation is shown to be dependent on host cross-dressed CD8α^+^ and CD103^+^ DCs^86^.

The emerging importance of the semi-direct pathway of allorecognition may provide a novel avenue for management of allograft rejection. The studies above highlight the importance of recipient DCs in alloreactive T-cell priming; the implication is that therapies which target host DCs may be of use in preventing rejection following transplantation. One example is the induction of high PD-L1 expression in DCs which results in diminished T-cell activation and prolonged graft survival^87^. However, the longevity of the semi-direct allorecognition pathway is not entirely clear. Evidence indicates that the direct allorecognition pathway is short-lived and its impact restricted to the initial few weeks after transplantation. Indirect allorecognition, by contrast, is longer-lived and relevant to late rejection responses^88–90^. If cross-dressed recipient DCs do indeed disappear shortly after transplantation, the functional significance of this pathway is not fully understood.

## Future perspectives

The rates of short-term graft survival have seen significant improvements in recent years. However, chronic rejection/allograft dysfunction continues to be a challenge, and certain estimates suggest that 25% of recipients may experience rejection by two years post-transplantation^91^. However, our improved understanding of the mechanisms of allorecognition may provide novel avenues for manipulation of alloresponses.

Current studies investigating the efficacy of ILC3-derived IL-22 in preventing GvHD have already reached human clinical trials. The regulatory activity of ILC2 cells also seems promising and suggests that expansion of this ILC subtype may be beneficial in transplant recipients. The discovery of the role of NK cells in graft rejection has prompted work assessing whether NK cell inhibition may offer a novel method of immunosuppression. Finally, the classification of a third pathway of allorecognition provides a new appreciation of the importance of recipient DCs in the alloresponse and highlights the potential for therapies that target this cell population. Although these new developments are compelling, a fuller understanding of how the pathways interact with the complete transplant response and confirmation of their replicability in humans is needed and will likely be the subject of future studies.
